# Supplementary material for: Triboelectric Charging Properties of the Functional Groups of Common Pharmaceutical Materials Using Density Functional Theory Calculations
Source: Pharmaceutics. 2024 Mar 21;16(3):433. doi: 10.3390/pharmaceutics16030433 (PMC10975866; doi:10.3390/pharmaceutics16030433)
Supplement: Supplementary file 1 [file pharmaceutics-16-00433-s001.zip › pharmaceutics-2919081-supplementary.pdf]

Supplementary materials

Triboelectric Charging Properties of the Functional Groups of Common Pharmaceutical Materials Using Density Functional Theory Calculations

James R. Middleton, Mojtaba Ghadiri and Andrew J. Scott

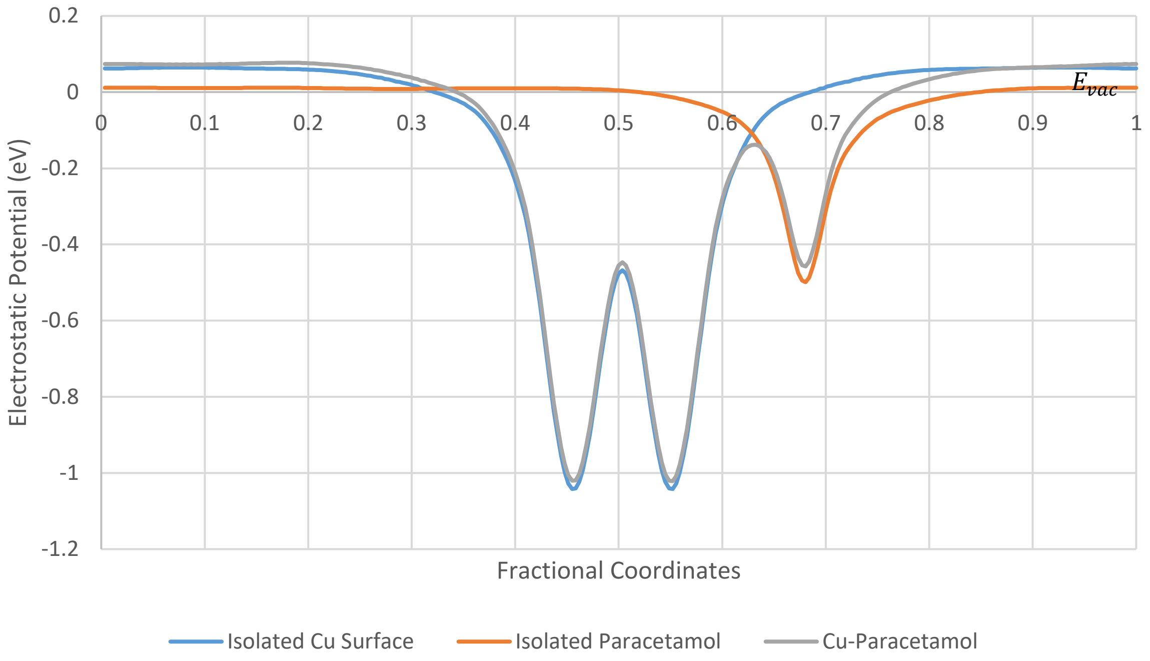

Figure S1. – Electrostatic potential of a copper (111) surface with a single paracetamol placed on its surface at its structurally optimised position. Electrostatic potential of the isolated surface and isolated molecule also shown.

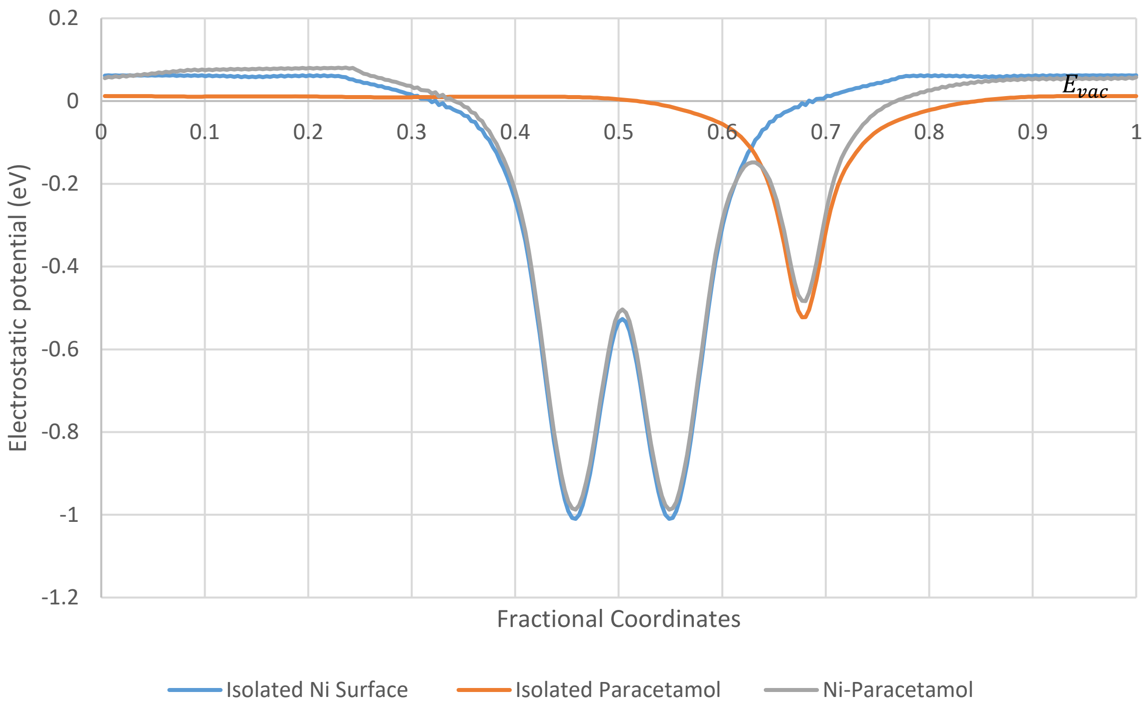

Figure S2. – Electrostatic potential of a nickel (111) surface with a single paracetamol placed on its surface at its structurally optimised position. Electrostatic potential of the isolated surface and isolated molecule also shown.

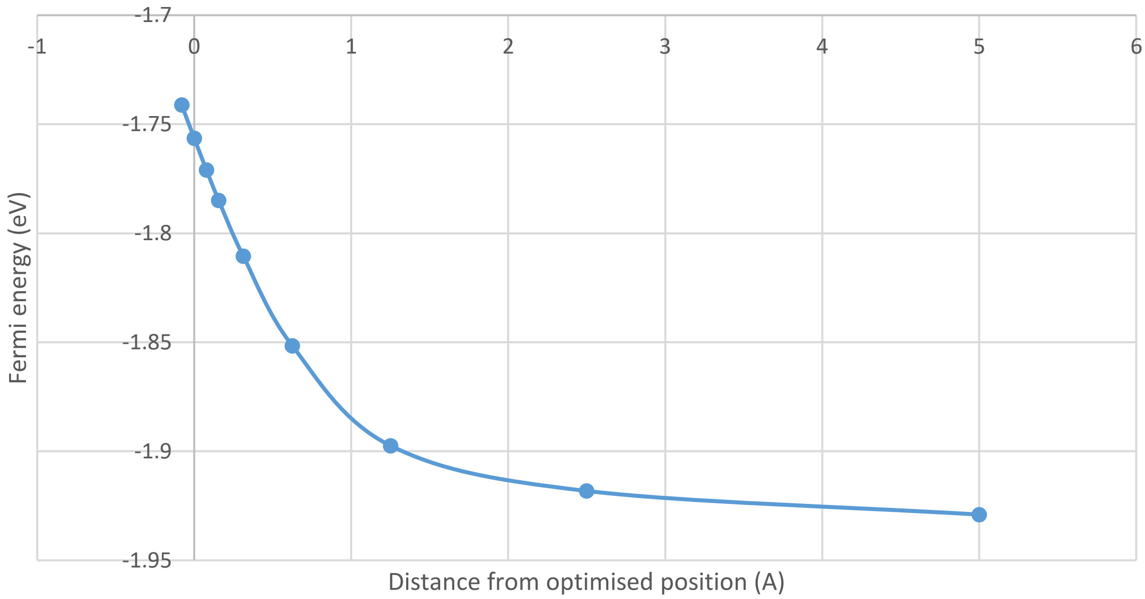

Figure S3. – The impact of the distance from the optimised position of the paracetamol molecule on the surface of the aluminium (111) system to the system Fermi energy.

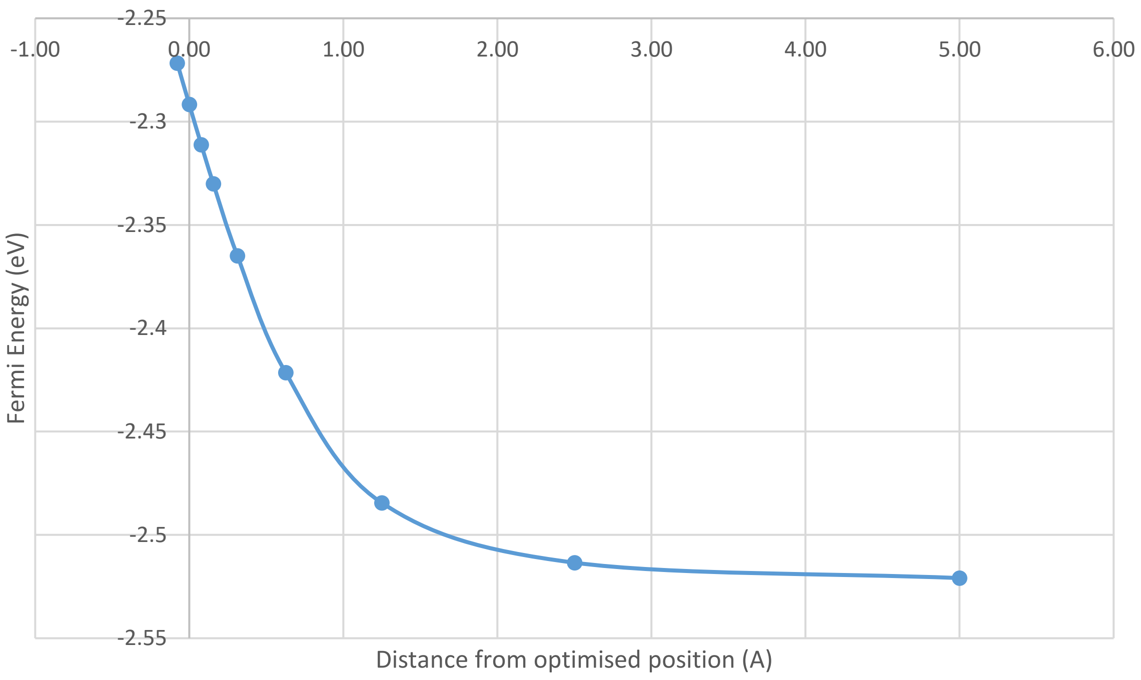

Figure S4. – The impact of the distance from the optimised position of the paracetamol molecule on the surface of the copper (111) system to the system Fermi energy.

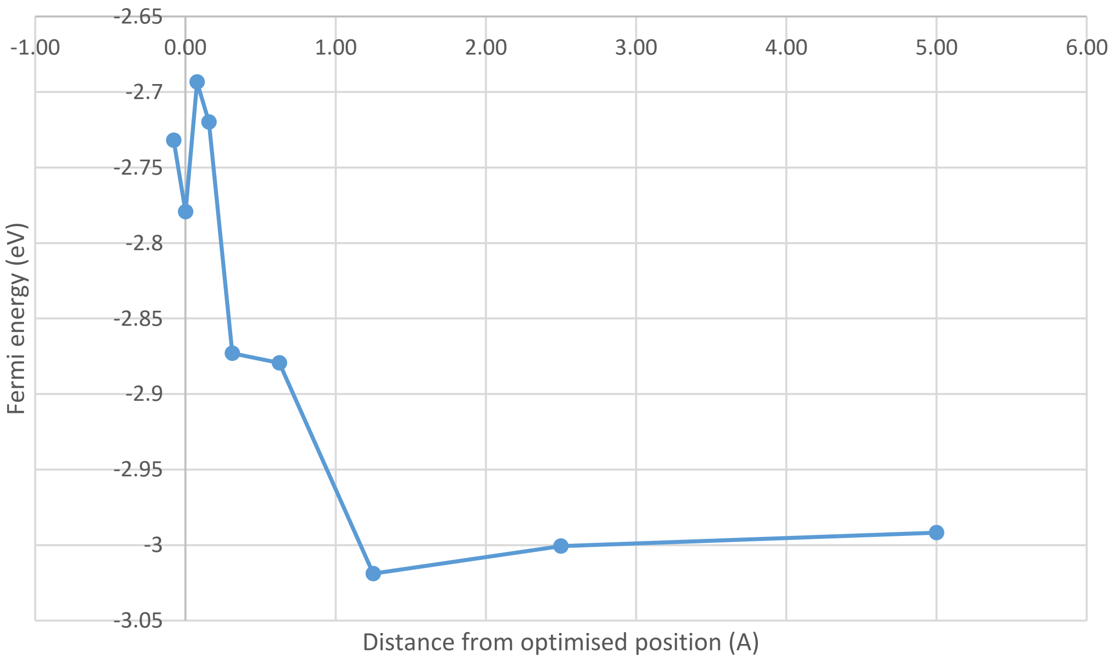

Figure S5. – The impact of the distance from the optimised position of the paracetamol molecule on the surface of the nickel (111) system to the system Fermi energy.

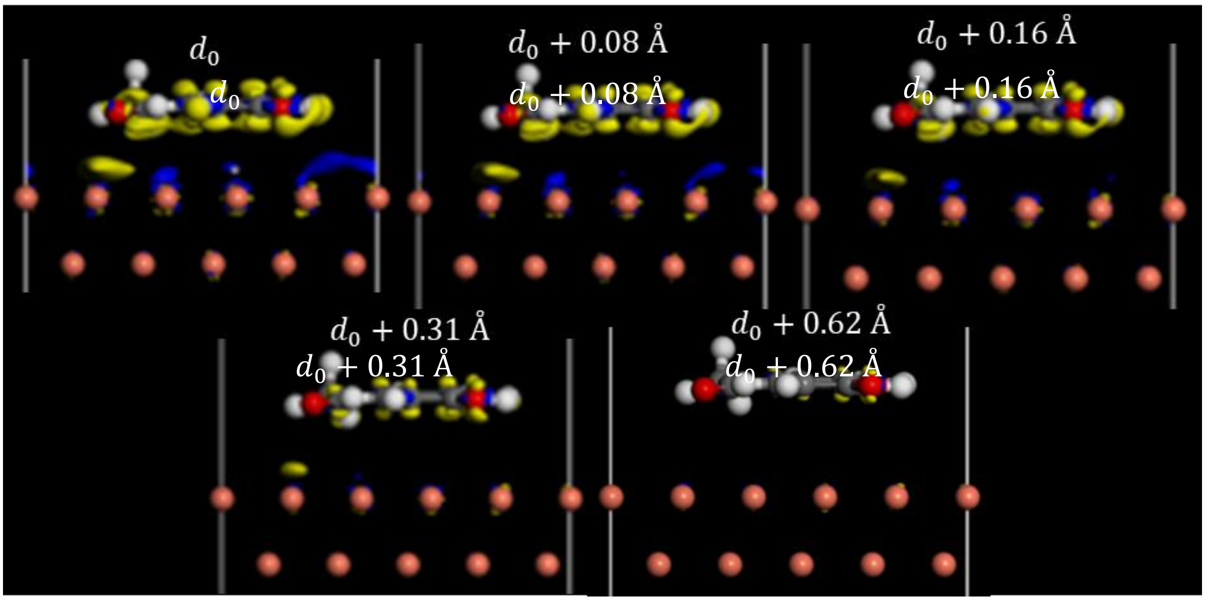

Figure S6. – Charge density difference iso surface of paracetamol on copper (111) surface at several distances. Side view. The geometry-optimised position of paracetamol is indicated by d_0. Iso value = $7x10^{-3}$ electron/Å^3.

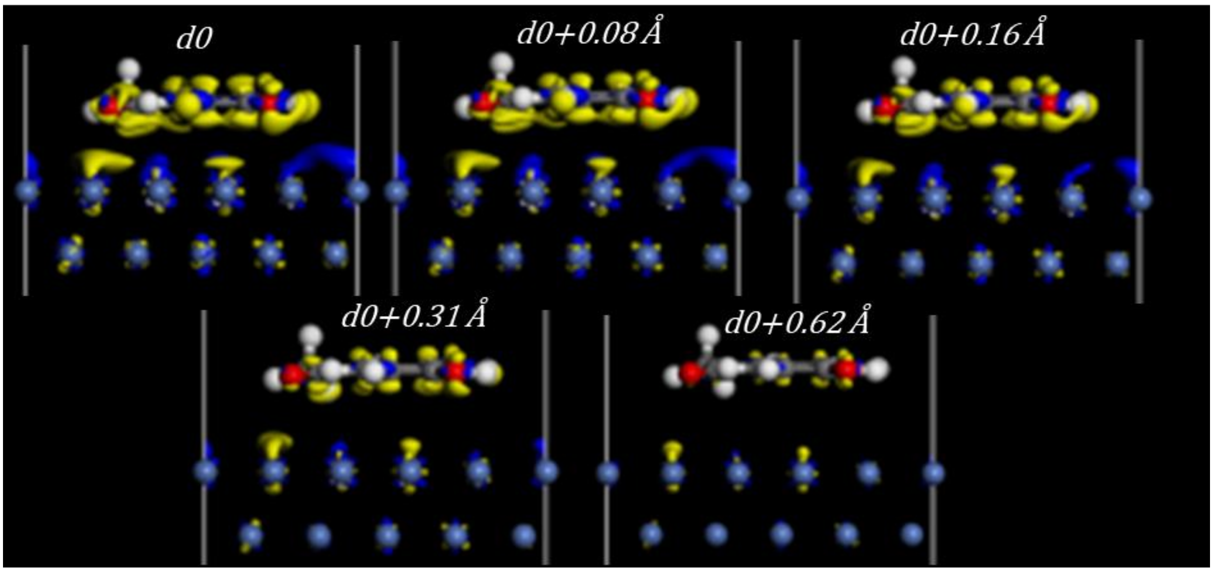

Figure S7. – Charge density difference iso surface of paracetamol on a nickel (111) surface at several distances. Side view. The geometry-optimised position of paracetamol is indicated by d_0 . Iso value = $7x10^{-3}$ electron/Å^3.
